# Electronic platform-based education for radiology residents: results of a two-year survey

**DOI:** 10.1186/s12909-023-04190-8

**Published:** 2023-03-30

**Authors:** Emilio Quaia

**Affiliations:** Department of Radiology, University of Padova, Via Giustiniani 2, Padova, 35128 Italy

**Keywords:** Education, Radiology, Resident, Electronic

## Abstract

**Background:**

Electronic platform based-learning for residents is increasing. The aim of this study was to identify the most reliable predictor variables related to the usage of electronic platform-based educational material for radiology residents which can predict a successful multiple-choice test during the academic year.

**Methods:**

A two year survey was conducted based on the records of electronic platform-based educational material for radiology residents. Radiology resident education was based on the educational content of two educational electronic platform databases named RADPrimer and STATdx (Elsevier, Amsterdam) consisting in evidence-based and expert-vetted summary information to support learning and diagnosing in radiology. A pool of multiple-choice questions was addressed in RADPrimer by each resident after 6 months from the beginning of each academic year, and at the end of the respective residency year as part of end of the year assessment. A per-resident analysis was performed to analyze the correlation between the amount of electronic platform content accessed by residents (measured by total login times, login frequency per month, and the number of per-topic addressed questions) in preparation for the electronic test during the academic year (predictor variables) and per-resident average percentage of correct answers on electronic test (outcome variable). Statistical significance (p < 0.05) was determined using logistic regression and correlation analysis.

**Results:**

Total login times (OR, 3; 95% CI, 2.2 -4), login frequency per month (OR, 4; 95% CI, 3.1–5.3), the number of per-topic addressed questions (OR, 3; 95% CI, 2.2 -4), and the number of topic-verified correct answers to multiple choice test (OR, 30.5; 95% CI, 12.8–80.9) all showed a statistically significant correlation with final percentage of correct answers on final year electronic test.

**Conclusion:**

The number of correct answers to multiple choice test was related to the number and frequency of login access, the number of per-topic addressed questions and the number of topic-verified correct answers to multiple choice test. The electronic-based educational material contributes significantly to a successful radiology residency program.

## Background

The primary goal of a residency program is to graduate prepared and professional radiologists who are able to add value to the health care team. Radiology departments must invest time and money to provide innovative educational opportunities [1].

Radiology residency education must evolve to meet the growing demands of radiology training. Traditionally, residents have been learning radiology through didactic frontal lectures and observational experience on extensive reading sessions. Radiology resident education in the era of COVID-19 was modified according to restrictions placed by health authorities, medical schools, and universities [2]. Consequently, traditional in-person teaching lectures, meetings, and conferences have been widely replaced by virtual live or recorded conferences [3]. Few published data are available investigating the radiology resident improvement in knoweldge based on innovative educational supports including electronic platform-based education.

We herby report the results of a two-year survey on radiology residents education based on electronic platform-based educational material, named RADPrimer and STATdx (Elsevier, Amsterdam), organized in different sections related to different educational topics of diagnostic radiology. The survey analysed multiple parameters, including the total number of logins and the frequency of logins per month to the electronic platforms, which are also related to the residents’ self-regulated learning ability as well as their online behavioral patterns .

The aim of the present study was to identify the most reliable predictor variables related to the usage of electronic platform-based educational material for radiology residents which can predict a successful multiple-choice test during the academic year.

## Materials and methods

### Structure of the local radiological residency

In Italy, the radiology residency program lasts four years with an increasing clinical responsibility of the resident through each year of curriculum. Beside frontal lectures, which correspond roughly to 10% of each resident time, all radiology residents undergo professional activity in diagnostic and interventional radiology sections for most of their time, besides informal curriculum related to attendance to scientific meetings and congresses [5] and attendance to multidisciplinary meetings.

### Overview of the investigated educational platforms

From the academic year 2019–2020, we obtained a combined subscription to two educational electronic databases named RADPrimer and STATdx consisting in supportive learning evidence-based information which were not freely accessible but only available to radiology residents as training radiology curriculum tool at University of Padova. Data on radiology resident survey data, based on RADPrimer and STATdx, are not publicly available, since both platforms are subscription based and usage data information from subscribers are private. Access to both plataforms is done via personal credentials for each resident. Usage data analysis was performed with resident name anonymization since personal information cannot be disclosed by the provider company (Elsevier) due to General Data Protection Regulation (GDPR) compliance rules. Resident personal data such as names and age were not included in data analysis, and each user was automatically assigned a numerical identification code for data collection and analysis. In accordance with relevant guidelines and regulations ethics approval was waived by ethics committee of Hospital of Padova since neither patient nor resident personal data were present in analysis. No patient data were included or uploaded in either platforms, since they contain evidence-based information based on peer-reviewed publications, with a detailed description of radiological features for each patology.

**Fig. 1:**
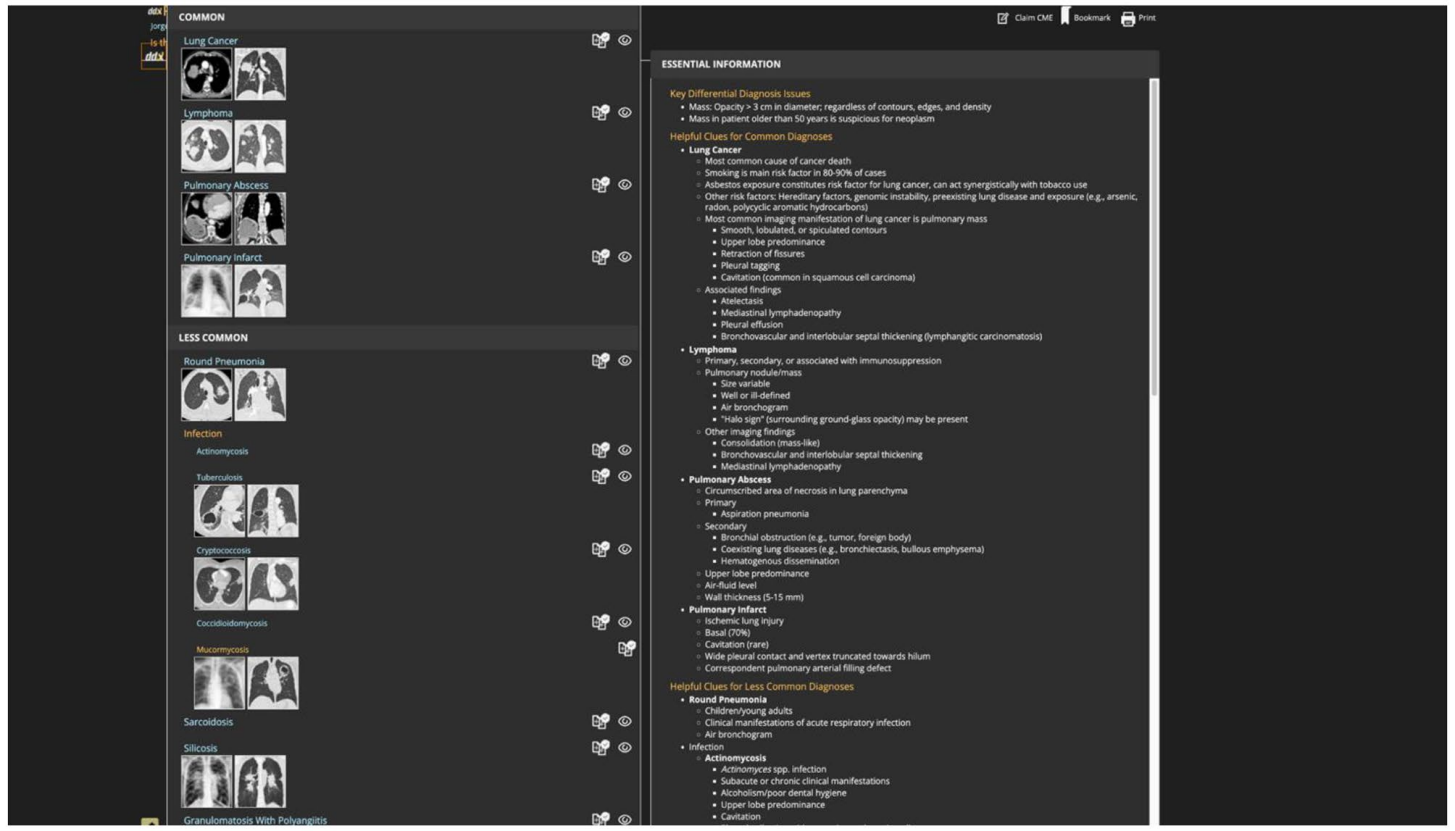
RADPrimer, picture of the platform

The implementation of these electronic educational platforms was not related to COVID outbreak since it was implemented before the pandemic and it was associated to planned frontal lectures which were administered consistently to residents during the academic year. RADPrimer is an educational e-learning platform with modules featuring case studies, reference images, and diagnosis solutions (Fig. 1). In RADPrimer there is a pool of questions to be addressed during the academic year after reading the content of each topic. STATdx is a diagnostic decision support system for radiologists which can be accessed by each resident or radiologist any time of the working day. STATdx provides instant access to the collective clinical experience and radiological knowledge of renowned sub-specialists in every field of radiology. Each radiology topic in RADPrimer and STATdx was crafted by a team of specialists, including leaders from prestigious institutions around the world, who wrote the content and selected each image.

**Fig. 2:**
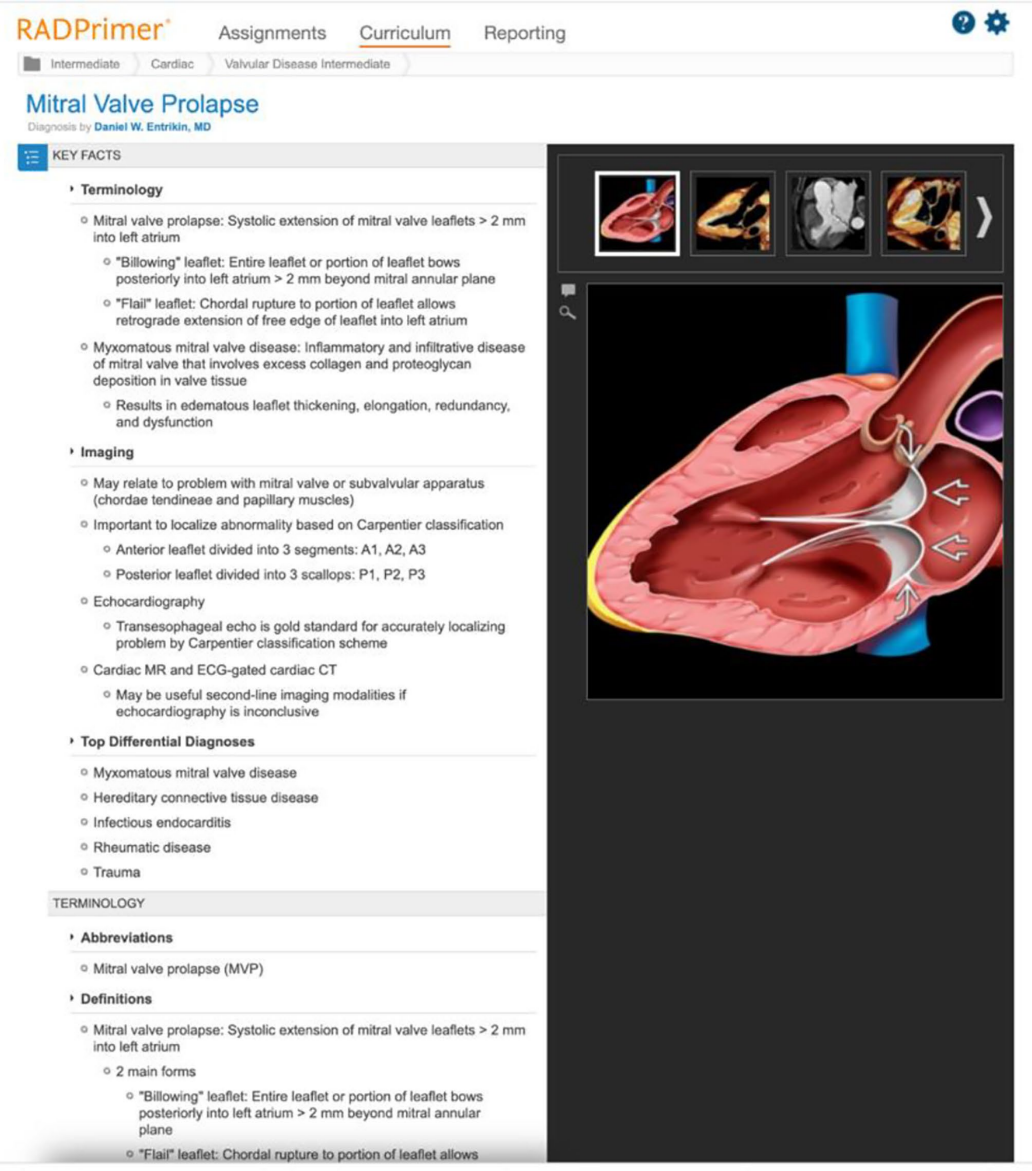
STATDX, picture of the platform

At the beginning of the academic year the RADPrimer educational content is provided to all residents according to the education topics matched to every residency year. Topics which are related to the school’s residency program were assigned to residents in relation to the year of training based on the curriculum-based residency program which was organized as follows: basic radiology knowledge during the first year of residency; musculoskeletal, urogenital, chest and gastro-abdominal radiology during the second year; neuroradiology and cardiac imaging during the third year; interventional, vascular and breast imaging during the fourth year of residency training. The electronic platform required residents to access e-learning platform by using personal credentials. As a fully online course, residents were required to study the e-platform material, take online multiple choice question test, and submit individual tasks for successful completion. Each radiology resident had to pass through all electronic assignments created by their faculty in preparation to the electronic final test. These assignments were elaborated by selecting lessons and associated case-based and traditional questions, from a pool of over 7.000 MCQs. Each lesson is embedded in a topic, containing an overview with a summary of lessons and respective learning objectives, as well as associated practice questions to help residents learn as they go along. All questions and lessons content were crafted by a team of educators and leading radiology experts at prestigious institutions around the world, based on peer-review published content. STATdx represents an even more extended electronic database and a continuous support for differential diagnoses during the reporting acivity of radiology residents (Fig. 2).

Multiple-choice questions (60 questions) were addressed in RADPrimer by each resident after 6 months from the beginning of each academic year, and at the end of the respective residency year as part of end of the year assessment. For each resident, an average percentage of correct answers to questions in preparation to the electronic test is provided at 6-month and at the end of the academic year. Finally, the average percentage of correct answers to multiple choice questions on 6-month and end of the year test is provided for each resident.

### Data analysis

We analysed data collected from January 2020 till January 2022. For this study, a total of 174 residents, including 80 residents in the academic year 2020 cohort − 25 residents in the first year, 24 residents in the second year, 17 residents in the third year, and 14 residents in the fourth year, and 94 residents in the academic year 2021 cohort − 35 residents in the first year, 24 residents in the second year, 17 residents in the third year, and 18 residents in the fourth year - were included. Residents’ total login time, login frequency, login regularity, number of per-topic addressed questions, number of electronic platform content access during the academic year, and final percentage of correct answers on final year electronic test were recorded.

### Statistical analysis

A logistic regression analysis was performed between number of electronic platform content access login including total login times, login frequency per month and number of per-topic addressed questions in prepartion to the electronic test (independent variables), and number of per-topic addressed questions in prepartion to the electronic test during the academic year (predictor variables) and per-resident average percentage of correct answers on electronic test (outcome variable). Statistical significance (p < 0.05) was determined using the Spearman correlation analysis.

## Results

There was a progressive increase in the login frequency number into the electronic platform content according to the year of residency with 902 ± 250 (mean ± SD) separate login access for the first year, 2276 ± 570 for the second year, 3106 ± 345 for the third year, and 2108 ± 550 for the fourth year.

Pre-test included a percentage of correct answers from 50 to 65%. The percentage of correct answers increased up to 80 − 97% on the first test taken after 6 months, and up to 87 − 99% on the final year test. The percentage of correct answers was higher for those residents who took pre and post-test assessment for each topic and for those who took an higher number of pre and post-tests during the academic year based on RADPrimer educational content provided at the beginning of academic year.

**Fig. 3:**
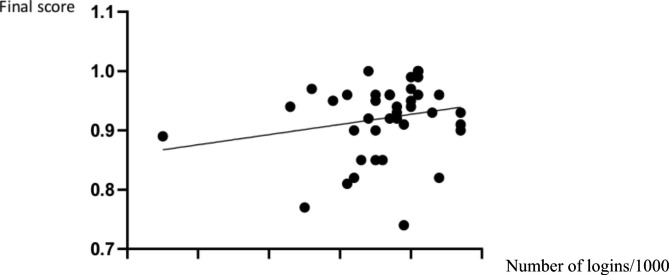
The figure shows the correlation results of the number of login times (normalized for 1000) to the number of correct questions on multiple choice question test

Subcategories that showed a statistically significant positive correlation with the final percentage of correct answers on multiple-choice question test are shown on Table 1. Spearman correlation was significant (ρ = 0.38; *P* < 0.05) for the number of login times and the number of correct question on the final year multiple choice question test (Fig. 3).

**Table 1:** Correlation and Odds Ratio (OR) between final test result and e-platform frequency

Independent Variables	OR (95% CIs) with final percentage of correct answers
*Total login time*	3 (2.2–4) *P* < 0.05
*Total login frequency per month*	4 (3.1–5.3) *P* < 0.05
*Per-topic addressed questions*	3 (2.2–4) *P* < 0.05
*Topics verified by multiple choice test*	30.5 (12.8–80.9) *P* < 0.05

## Discussion

The primary aim of a radiology residency program is to train residents to become well-educated, independent, professional, and skilled radiologists [4]. We aimed to do a single-center retrospective study to identify the most reliable predictor variables related to the usage of electronic platform-based educational material for radiology resident to predict a successful final year multiple choice test.

We found that number of total login times, login frequency per month, number of per-topic addressed questions, and number of topic-verified correct answers to multiple choice test all showed a positive correlation with the number of correct answers to multiple choice test. We added electronic platform – based educational material to the formal curriculum to provide teaching material for all subjects of the radiological residency which were not covered by local lectures. Our results seem to confirm the importance of electronic scores in multiple choice question tests and, most likely, in all professional life of radiologists.

Electronic-based educational material provides several advantages over frontal lectures including the possibility to access educational material from virtually any site that has a computer and an internet connection and at virtually any time of the day or night [5], to integrate text, images, and sounds in ways that standard textbooks cannot, to access and customize educational materials for their own learning preferences [6], and the greater degree of interaction they permit. Electronic educational material can be presented through electronic didactic sheets and in multiple-choice test format that helps learners evaluate their performance and identify their own strengths and weaknesses and make teachers aware of their progress in knowledge.

Electronic-based educational material is widely used in educational sciences, including medical education courses. This paradigm shift challenges the tradition of lectures and shifts the educational experience in a learner-centred [7] way since each resident is empowered to organize his own curriculum based on electronic materials instead of frontal lectures. This shift was pushed even further in radiology education by the COVID-19 pandemic [8] with use of advanced digital e-learning strategies, such as microlearning and visual learning tools [9]. At a time when usual active learning practices have been stopped or, at least, changed, trainees have found electronic-based educational platform a very useful online learning tool to keep their learning going.

Our study provides incremental data to the existing literature that offers insight into factors that contribute to a successful radiology residency program based on e-learning educational tools. According to a previous survey [10] which included 200 radiology residents in USA, the average radiology resident works 50 h per week excluding time on call (range, 40–60 h) and 67 h per week including time on call. Residents at all levels spent an average of 9 h per week studying with a range from 2 to 27 h per week. Most of that time was spent reading textbooks (4–5 per year), followed by journals (mainly during the third and fourth year of residency), notes and teaching files. Residents spent an average of 7 h per week attending lectures and conferences. Beside the changes in didactic flow due to COVID outbreak, regardless of how effective electronic-based material is, frontal lecture should not be entirely supplanted. The numerous weekly hours dedicated to the assistance activity by residents often do not allow adequate coverage of the hours of frontal teaching required by the program. The use of an electronic-based educational platform, allows the availability of a vast network of information that can potentially partially replace the traditional frontal lectures.

The use of electronic-based educational platform allows immediate and exhaustive access to the essential information necessary to correctly propose a differential diagnosis relating to the radiological findings identified during the evaluation of the images and described in the report. In addition, electronic-based educational platform allows learners to conduct a personal test to evaluate the degree of self-learning achieved within the various teaching modules and verify the increase in knowledge in the various areas of imaging. This was achieved by the constant and immediate access to more detailed information relating to the different pathological findings of each disease detectable by imaging techniques, and formulation of the main differential diagnoses for each pathological finding. Our results are in keeping with previous studies which confirm the value of e-learning tools to improve knowledge of radiology residents both using conventional e-learning tools such as Radiopaedia, Auntminnie, or Eurorad [11], blended learning, flipped learning, digital teaching files [12], or even more sophisticated e-learning tool including virtual and augmented reality [13].

Interactive learning is crucial for effective medical, and particularly radiology education, since it provides active retention of information [14, 15]. In particular, the use of multiple-choice questions contributes to develop a critical thinking which is essential in the clinical practice. Students must take time to apply concepts, already consolidated, to answer multiple-choice questions even during recorded lectures. Multiple-choice questions will invariably lead to a higher level of learning concepts and solicitate learners to read more about a specific topic [16]. The use of electronic-based educational platform provides a complete coverage of the whole theoretical knowledge which otherwise could not have been covered by the lectures only and allows radiology residents to have basic knowledge in all areas of radiology diagnostics, from a theoretical point of view. Previously, it was impossible to cover such a vast amount of information based solely on the lectures or on the study of traditional texts.

## Conclusions

In conclusion, the number of correct answers to multiple choice test was related to the number and frequency of login access, the number of per-topic addressed questions and the number of topic-verified correct answers to multiple choice test. The electronic-based educational material contributes significantly to a successful radiology residency program.

## Data Availability

The data that support the findings of this study are at https://app.radprimer.com/faculty/assignments but restrictions apply to the availability of these data, which were used under license for the current study, and so are not publicly available. Data are however available from the authors upon reasonable request and with permission of Elsevier.
